# Self-objectification during the perinatal period: The role of body surveillance in maternal and infant wellbeing

**DOI:** 10.21203/rs.3.rs-2714781/v1

**Published:** 2023-03-21

**Authors:** Lauren M. Laifer, Olivia R. Maras, Gemma Sáez, Sarah J. Gervais, Rebecca L. Brock

**Affiliations:** University of Nebraska-Lincoln; Arizona State University; Universidad de Extremadura; University of Nebraska-Lincoln; University of Nebraska-Lincoln

**Keywords:** self-objectification, pregnancy, perinatal period, objectification theory, body surveillance, body image, depression, mother-infant bonding, infant socioemotional functioning

## Abstract

Pregnancy represents a unique time during which women’s bodies undergo significant physical changes (e.g., expanding belly, larger breasts, weight gain) that can elicit increased objectification. Experiences of objectification set the stage for women to view themselves as sexual objects (i.e., self-objectification) and is associated with adverse mental health outcomes. Although women may experience heightened self-objectification and behavioral consequences (such as body surveillance) due to the objectification of pregnant bodies in Western cultures, there are remarkably few studies examining objectification theory among women during the perinatal period. The present study investigated the impact of body surveillance, a consequence of self-objectification, on maternal mental health, mother-infant bonding, and infant socioemotional outcomes in a sample of 159 women navigating pregnancy and postpartum. Utilizing a serial mediation model, we found that mothers who endorsed higher levels of body surveillance during pregnancy reported more depressive symptoms and body dissatisfaction, which were associated with greater impairments in mother-infant bonding following childbirth and more infant socioemotional dysfunction at 1-year postpartum. Maternal prenatal depressive symptoms emerged as a unique mechanism through which body surveillance predicted bonding impairments and subsequent infant outcomes. Results highlight the critical need for early intervention efforts that not only target general depression, but also promote body functionality and acceptance over the Western “thin ideal” of attractiveness among expecting mothers.

## Introduction

Self-objectification—seeing the self as a sexual object—has been recognized as an important contributor to women’s mental health since the phenomenon was formally introduced to the psychological literature in the form of objectification theory two and a half decades ago (Fredrickson & Roberts, 1997; Roberts et al., 2018). According to this framework, by living in a culture in which women are commonly reduced to their bodily appearance, women learn to view their bodies from a third person’s perspective (i.e., self-objectify, Fredrickson & Roberts, 1997) and often engage in persistent body surveillance (McKinley & Hyde, 1996). Further, many women feel pressure to fit cultural ideals of attractiveness and may experience body shame and dissatisfaction if their bodies do not align with these often-unattainable standards (McKinley & Hyde, 1996; Tiggemann & Lynch, 2001).

Self-objectification and its behavioral consequences, such as body surveillance, set the stage for adverse mental health outcomes that disproportionately affect women (e.g., anxiety, depression, eating disorders; Fitzsimmons-Craft & Bardone-Cone, 2012; Jones & Griffiths, 2015; Roberts et al., 2018; Rubin & Steinberg, 2011; Sun, 2018) and can interfere with parenting and child wellbeing (Chapman et al., 2021; Deave et al.,2008; Galbally & Lewis, 2017; Herba et al., 2016). The current study presents a novel conceptual framework in which self-objectification, as manifested by persistent body surveillance, is significantly linked to maternal mental health during pregnancy (i.e., body dissatisfaction, depression) and undermines infant socioemotional functioning through impaired mother-infant bonding following childbirth.

## Objectification Theory And The Consequences Of Self-objectification

Objectification theory posits that “*women are most targeted for objectification during their years of reproductive potential*” (Fredrickson & Roberts, 1997, p. 192). Indeed, objectification (reduction to appearance and sexual body parts; loss of autonomy; denial of subjectivity) is heightened during key stages in which girls and women undergo physical changes (e.g., puberty), and it may also be heightened during pregnancy. Specifically, pregnant bodies become “public property,” with people looking at, commenting on, and even touching the bodies of pregnant women (Kukla, 2005). Further, women may experience increased body surveillance and related body dissatisfaction across pregnancy and postpartum as their bodies become more removed from a potentially internalized “thin ideal” of attractiveness. These bodily changes may also be connected to other facets of self-objectification (Talmon & Ginzburg, 2016), such as feeling like their autonomy and freedoms are restricted (Sutton et al., 2011). Women may feel like their pregnant bodies have become hyper-visible, while other aspects of their personhood have been rendered invisible.

Indeed, a systematic review of research on self-objectification and motherhood by Beech and colleagues (2020) revealed that self-objectification among mothers is associated with a range of negative outcomes, such as difficulties breastfeeding, fear of childbirth, depression, and disordered eating. Despite these possibilities, remarkably few studies have examined whether the tenets of objectification theory apply to the perinatal period (Beech et al., 2020; Brock et al., 2021; Rubin & Steinberg, 2011). Because of the significant changes that women’s bodies undergo during pregnancy and postpartum (e.g., expanding belly, larger breasts, weight gain), the present investigation focused on body surveillance (see Talmon & Ginzburg, 2016, for other important facets of self-objectification). We posit that bodily changes during pregnancy and concomitant increases in objectification from others may cause women to engage in more persistent body surveillance and experience associated mental health problems (e.g., body dissatisfaction, depression). These decrements in mental health may, in turn, undermine the quality of mother-infant interactions (see McNamara et al., 2019 for a review).

Increasingly, researchers have examined whether markers of self-objectification among mothers, such as body surveillance, and associated mental health consequences spill over into parenting and child development. Although much of this research has focused on women with adolescent children (e.g., Arroyo & Andersen, 2016; Katz-Wise et al., 2013), research also provides evidence for the intergenerational transmission of body dissatisfaction and disordered eating behaviors in younger children (Rodgers et al., 2013; Spiel et al., 2012). For example, maternal body dissatisfaction is prospectively associated with lower child body esteem in middle childhood (Rodgers et al., 2020). Maternal body dissatisfaction has also been linked to the use of more controlling feeding practices (e.g., food restriction, pressure to eat) with preschool-age children (Blissett & Haycraft, 2011; Duke et al., 2004; Rodgers et al., 2013; Webb & Haycraft, 2019), which may interfere with children’s regulatory capacities by teaching them to view eating as a primary strategy for emotion regulation (Farrow et al., 2015).

Despite growing evidence that body surveillance in mothers and associated mental health consequences (e.g., depression, body dissatisfaction) may negatively impact children, comparatively less is known about the impact of body surveillance on infant socioemotional functioning. Thus, we extend these considerations to examine whether body surveillance impacts not only maternal mental health, but also infants by undermining mother-infant bonding following childbirth. We posit that increased body surveillance, resulting from a culture that persistently objectifies women’s bodies, is linked to maternal mental health concerns and the likelihood that mothers experience difficulties bonding with their infants.

## Body Surveillance and Body Dissatisfaction During Pregnancy

Research on body dissatisfaction during pregnancy has demonstrated mixed findings (Coker & Abraham, 2015; Fuller-Tyszkiewicz et al., 2020; Loth et al., 2011; Skouteris et al., 2005). Presumably, there are important individual differences in how women experience their body transformation across pregnancy. Some women may embrace this transformation and become more appreciative of what their bodies are physically capable of – nurturing and supporting a developing fetus (Rubin & Steinberg, 2011). This appreciation of body functionality, in turn, may buffer against distress related to the rapid physical changes that occur across pregnancy and postpartum (Clark et al., 2009). Alternatively, some pregnant women might be more susceptible to societal pressures around their bodies and continue to hold their bodies to unrealistic beauty standards, focusing more on body image than functionality (Johnson et al., 2004). For instance, some mothers may aspire to gain minimal gestational weight and to return to their pre-pregnancy figure, or “bounce back,” quickly after childbirth (Watson et al., 2015). Perceived sociocultural pressure to remain thin is associated with maternal distress and body dissatisfaction during pregnancy and the postpartum (Dryer et al., 2020; Fuller-Tyszkiewicz et al., 2013; Kamysheva et al., 2008; Lovering et al., 2018). Further, gaining less than the recommended amount of weight during pregnancy is associated with higher risk of preterm birth and low birthweight (Han et al., 2011), which predict self-regulatory difficulties as early as infancy (Arpi & Ferrari, 2013).

## Body Surveillance and Depression During Pregnancy

Body surveillance is associated with higher levels of depressive symptoms during the perinatal period (Rodgers et al., 2018; Rubin & Steinberg, 2011). These associations are alarming given that pregnant women are already at increased risk for depression during the perinatal period, with one in five women endorsing depressive symptoms across pregnancy and postpartum (Underwood et al., 2016; Woolhouse et al., 2015). In the United States, over half of women with perinatal depression go undetected, undiagnosed, and untreated for this condition (Cox et al., 2016). This represents a significant public health burden given that antenatal depression contributes to the proliferation of a range of mental health concerns in both parents and children (Hentges et al., 2019; Waters et al., 2014).

## Maternal Mental Health And Infant Development

Maternal psychopathology during pregnancy, particularly depression, is a robust predictor of poor child outcomes, including increased risk for child psychopathology (Barker et al., 2011; Goodman et al., 2011; Goodman & Gotlib, 1999; Szekely et al., 2021). Indeed, perinatal depression predicts socioemotional difficulties (e.g., crying for long periods of time) as early as infancy (Field, 2017; Porter et al., 2019). Mother-infant bonding (i.e., the emotional tie between mother and infant; Bicking Kinsey & Hupcey, 2013), is a salient mechanism through which depression can undermine child functioning (Lefkovics et al., 2014; Slomian et al., 2019). In particular, bonding during the first 6 months postpartum is critical to infant socioemotional development, as infants largely depend on their caregivers to regulate their emotions (Rosenblum et al., 2009), and early mother-infant bonding impairments predict infant socioemotional difficulties as early as 6-months postpartum (Ramsdell & Brock, 2021). Women who report higher levels of depression during and after pregnancy tend to demonstrate greater impairments in mother-infant bonding (Moehler et al., 2006; Nonnenmacher et al., 2016; O’Higgins et al., 2013), and research suggests that negative cognitions associated with perinatal depression may undermine maternal motivation to bond with the infant following childbirth (Muzik & Borovska, 2011).

Although maternal depression is a robust predictor of bonding impairments and associated infant maladjustment, researchers also posit that body dissatisfaction during pregnancy impacts mothers’ developing bonds with their infants and, subsequently, child socioemotional functioning (Bergmeier et al., 2020). Indeed, the physical changes that occur over the course of pregnancy–and how these changes are perceived and experienced–represent one of the first ways in which mothers interact with their babies. Women who embrace the bodily changes associated with pregnancy may be more likely to engage emotionally with their babies prior to childbirth, whereas women who feel negatively about these changes and experience greater body dissatisfaction may face more bonding difficulties (Kirk & Preston, 2019). Further, some women may experience a loss of agency and control over their own bodies during pregnancy (Kinloch & Jaworska, 2021). This perceived loss of control, which is associated with maternal distress (Hodgkinson et al., 2014), might also interfere with antenatal attachment.

## The Present Study

**Figure 1:**
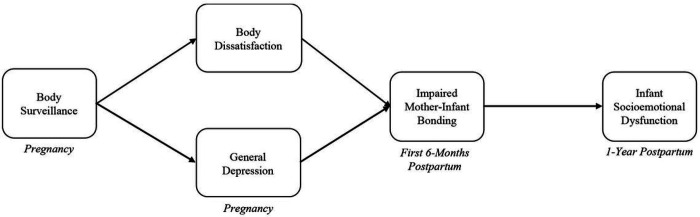
Novel Conceptual Model Linking Body Surveillance During Pregnancy to Infant Socioemotional Dysfunction at Age One

Objectification theory would suggest that women may be at heightened risk for self-objectification during pregnancy, which can compromise their mental health, and past research suggests a robust link between maternal mental health and bonding difficulties. Taken together, this work suggests that elevations in self-objectification and its correlates (e.g., body surveillance, body dissatisfaction, depression) during pregnancy might ultimately undermine healthy infant socioemotional development. Building on recent work applying objectification theory to motherhood (e.g., Beech et al., 2020), we present a novel conceptual framework (see Fig. 1) in which mothers who report greater body surveillance during pregnancy–a marker of self-objectification–experience higher levels of prenatal depressive symptoms and body dissatisfaction that, in turn, uniquely predict greater mother-infant bonding impairments following childbirth, thereby undermining infant socioemotional functioning at age 1. An integration of research and theory in the areas of objectification and maternal-infant health has the potential to impact both maternal and infant wellbeing by identifying largely overlooked intervention targets during pregnancy (i.e., body surveillance and body dissatisfaction) that arise as a consequence of living in a culture of persistent objectification.

## Method

### Participants and Procedures

The present study is part of a multi-method, longitudinal study examining how couples navigate the transition from pregnancy to postpartum; thus, participants also completed other procedures beyond the scope of the present study. All participants identified as cisgender upon study entry. Most women were in the second (38.4%) or third (58.5%) trimester of pregnancy. On average, there was one child living at home during pregnancy (*SD*= 1.18); more than half of women (57.9%) had no children and were experiencing the transition into parenthood for the first time. The majority of women were married (84.9%). Annual household income ranged from less than $9,999 to more than $90,000, with a median household income of $60,000 to $69,999. Nearly half (47.8%) reported earning $50,000 to 59,999 or less which converges with federal guidelines for defining low-income status (Roberts et al., 2012). Reflecting the Midwestern region where the study was conducted, women were primarily White (89.3%), and 9.4% identified as Hispanic or Latina. On average, women were 28.67 years of age (*SD* = 4.27), and most women were employed at least 16 hours per week (74.2%). Modal education was a bachelor’s degree (46.5%). During follow-up assessments, it was determined that one infant was diagnosed with trisomy 21, and one mother experienced a miscarriage. As such, those families were excluded from analyses to focus on women with typically developing infants (50% male) for a final sample of 157 perinatal women.

There were four waves of data collection spanning February 2016 to April 2019. To address the aims of the present study, we assessed body surveillance, body dissatisfaction, and depressive symptoms using self-report questionnaires administered to mothers during the appointment. We assessed mother-infant bonding at 1-month postpartum (*M* = 1.12 months, *SD* = 0.29) and 6-months postpartum (*M* = 6.32 months, *SD* = 0.36) using a self-report questionnaire. Additionally, when the infant turned 1year of age (*M* = 12.80 months, *SD* = 0.76), both parents reported on infant socioemotional dysfunction. All procedures were approved by the University of Nebraska-Lincoln Institutional Review Board.

### Measures During Pregnancy

#### Body Surveillance.

The Body Surveillance subscale of the Objectified Body Consciousness Scale (OBCS, (McKinley & Hyde, 1996) was used to assess body surveillance, an important manifestation of self-objectification. During pregnancy, mothers rated the degree to which they persistently monitored their bodily appearance on a scale from one (*strongly disagree*) to six (*strongly agree*), with a *not applicable* option (coded as missing) for items that did not apply. The Body Surveillance subscale contains 8 items, including “During the day, I think about how I look many times” and “I rarely worry about how I look to other people” (reverse coded). Items were averaged with higher scores indicating more body surveillance (Cronbach’s α = .85).

#### Depression.

Maternal depressive symptoms were assessed using the General Depression subscale of the Inventory of Depression and Anxiety Symptoms (IDAS-II; Watson et al., 2012). The IDAS-II is a 99-item self-report questionnaire designed to assess general and specific symptom dimensions of depression and related anxiety disorders. Participants rated their feelings and experiences during the past two weeks on a scale from 1 (*not at all*) to 5 (*extremely*). The general depression subscale consists of 20 items (e.g., “I felt inadequate,” “I felt discouraged about things”), with possible scores ranging from 20 to 100 (Cronbach’s α = 0.84).

#### Body Dissatisfaction.

The Eating Pathology Symptoms Inventory (EPSI; Forbush et al., 2013, 2014) was used to assess body dissatisfaction reported by mothers during pregnancy. The EPSI is a factor analytically derived scale of eating disorder (ED) symptoms. The Body Dissatisfaction subscale consists of 7 items (e.g., “I did not like how clothes fit the shape of my body,” “I wished the shape of my body was different”) and captures the higher-order, shared dimension among ED symptoms. Participants rated how frequently each statement applied to them during the past month on a scale from 0 (*never*) to 4 (*very often*). Items responses were summed, with possible scores ranging from 0 to 28 (Cronbach’s α = 0.88).

### Measures At 1- And 6-months Postpartum

#### Impaired Mother-Infant Bonding.

Postpartum mother-infant bonding was assessed using the Postpartum Bonding Questionnaire (PBQ; Brockington et al. 2001). The PBQ is a 25-item, factor-analytically derived, parent-report measure of a parent’s feelings or attitudes toward their baby. The PBQ assesses impaired bonding, rejection and anger, anxiety about care, and risk of abuse, represented as four subscales that can be summed for a total score. Participants rated their agreement with a series of statements on a 6-point Likert scale. Positive responses (e.g., “I feel close to my baby”) were scored from 0 (*always*) to 5 (*never*), while negative responses (e.g., “My baby irritates me”) were scored from 0 (*never*) to 5 (*always*). Items were summed to generate a total score, with low scores denoting good bonding and high scores indicating impaired bonding. Scores at 1- and 6-months postpartum were internally consistent (Cronbach’s α = 0.88 at 1 month and Cronbach’s α = 0.86 at 6 months). Scores at each time point were highly correlated (*r* = .76, *p* < .001) and were thus aggregated to provide a robust measure of mother-infant impaired bonding during the first 6 months postpartum.

### Measures At 1-year Postpartum

#### Infant Socioemotional Dysfunction.

The Ages and Stages Questionnaire: Social-Emotional, Second Edition (ASQ:SE-2; (Squires et al., 2015) was used to assess socioemotional dysfunction when the infant turned one year of age. Participants reported how frequently their infant had engaged in a series of behaviors (e.g., “Smiles at you and family members?”, “Cries for long periods of time?”) using the following scale: *often or always* (score = 1), *sometimes* (score = 5), and *rarely or never* (score = 10). They were also asked to indicate *if this is a concern* (score = 5). Items were aggregated to obtain an overall score ranging from 0 to 345 (reverse coding items that represent competencies), with higher scores indicating greater infant socioemotional dysfunction. The correlation between maternal and paternal reports was significant (*r* = .33, *p* < .001). Therefore, scores were aggregated to obtain a score of infant socioemotional dysfunction based on multiple parental reports to produce a less biased and more reliable estimate (Lengua et al., 2008). The ASQ:SE-2 has demonstrated good reliability and validity, and there was adequate internal consistency in the present sample (Cronbach’s α = .71).

### Data Analytic Plan

We tested a series of mediation models in Mplus 8.0. (Muthén & Muthén, 2010). Missing data were addressed with full information maximum likelihood estimation (covariance coverage ranged from .74 to 1.00), which retains all participants and is preferred over more traditional approaches for handling missing data that introduce bias (e.g., pairwise deletion; Enders, 2010). A series of demographic characteristics (e.g., maternal age, relationship duration, first-time parenthood status, minority racial/ethnic identity, and low-income status) were screened for potential inclusion as control variables. First-time parenthood status was associated with mother-infant bonding and was therefore included as a control. We also controlled for week of pregnancy when the initial assessment occurred to account for differing time intervals between the pregnancy and follow-up assessments across participants.

Mediation models were just-identified . To test for mediation, a nonparametric resampling method (bias-corrected bootstrap) with 10,000 resamples was performed to derive the 95% confidence intervals for indirect effects (Preacher et al., 2007). Bias-corrected bootstrapped confidence intervals were used to determine significance of effects given they are robust to violations of univariate and multivariate normality. Data management and analysis procedures for this project were registered (https://osf.io/hprk8), and we made no deviations from that plan. Because we had prior knowledge of data from this longitudinal study, we did not preregister study hypotheses.

## Results

Descriptive statistics and correlations are reported in Table 1. As expected for a community sample, levels of body surveillance, general depression, and body dissatisfaction in mothers during pregnancy were relatively low, as were impairments in bonding during the first 6 months postpartum and infant socioemotional dysfunction at 1-year postpartum. There was a large correlation between body surveillance and body dissatisfaction during pregnancy (*r* = .54, *p* < .001), as well as a moderate correlation between body surveillance and general depression (*r* = .30, *p* < .001). Body surveillance in mothers was significantly correlated with impaired bonding during the first 6 months postpartum (*r* = .16, *p* < .05). There was a moderate correlation between general depression and body dissatisfaction during pregnancy (*r* = .34, *p* < .001). Small but significant correlations between general depression and impaired bonding (*r* = .26, *p* < .001) and between body dissatisfaction and impaired bonding (*r* = .23, *p* < .01) emerged. Last, impaired bonding was associated with greater infant socioemotional difficulties (*r* = .21, *p* < .01).

### Mediation Model With General Depression As Critical Mediator

**Figure 2:**
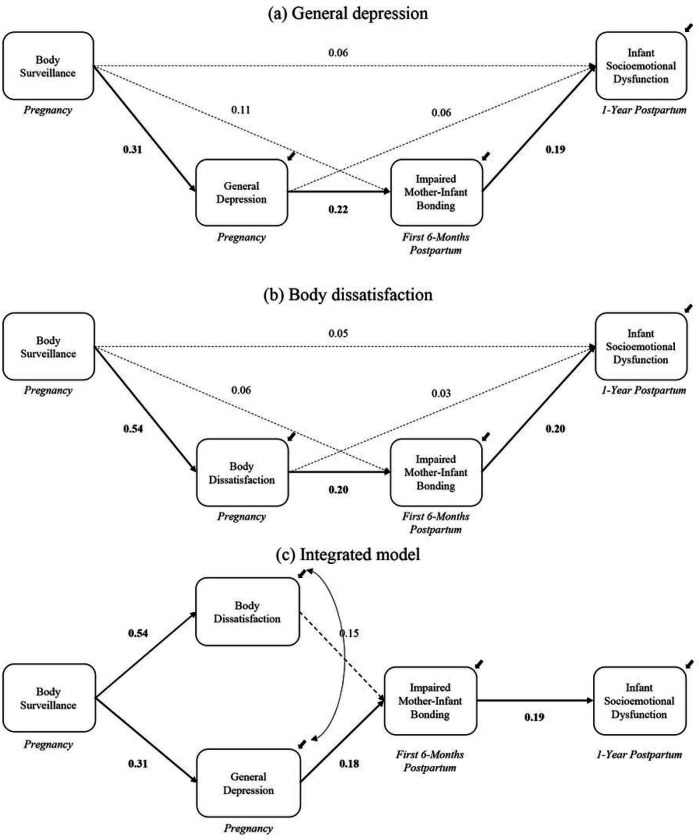
Results of Path Analysis Note. Standardized coefficients are reported. Significant coefficients are bolded and are depicted by solid lines. Bias-corrected confidence intervals (CIs) based on 10,000 bootstrapped samples were calculated to determine significance of effects. If a CI did not contain zero, the effect was significant. Significant indirect effects are depicted by solid lines. In figure 2(a), the overall indirect effect of body surveillance on infant socioemotional dysfunction through maternal general depression and impaired mother-infant bonding was significant, 95% CI [.04, .59]. In figure 2(b), the overall indirect effect of body surveillance on infant socioemotional dysfunction through maternal body dissatisfaction and impaired mother-infant bonding was significant, 95% CI [.03, 1.00]. Finally, in figure 2(c), the overall indirect effect of body surveillance on infant socioemotional dysfunction at one year through maternal general depression and impaired mother-infant bonding was significant, 95% CI [.03, .55]. All direct paths were tested in the integrated model (i.e., body surveillance → impaired bonding; body surveillance → socioemotional dysfunction; body dissatisfaction → socioemotional dysfunction; depression → socioemotional dysfunction) but were not significant and are not depicted in the figure. Please refer to Table 2 for full model results.

First, we tested a serial mediation model with body surveillance → *general depression* → impaired mother-infant bonding → infant socioemotional dysfunction. Full model results are reported in Table 2 and Fig. 2a. Greater body surveillance was associated with greater general depression during pregnancy, 95% CI [1.29, 4.12]. Further, greater maternal depression predicted higher levels of impaired mother-infant bonding over the first 6 months postpartum, 95% CI [.05, .32]. In turn, bonding difficulties predicted greater socioemotional dysfunction for infants at 1 year of age, 95% CI [.04, .77]. The overall indirect effect of body surveillance on infant socioemotional dysfunction through maternal general depression and impaired mother-infant bonding was significant, 95% CI [.04, .59].

### Mediation Model With Body Dissatisfaction As Critical Mediator

Next, we tested a serial mediation model with body surveillance → *body dissatisfaction* → impaired mother-infant bonding → infant socioemotional dysfunction. Full model results are reported in Table 2 and Fig. 2b. Greater body surveillance was associated with greater body dissatisfaction during pregnancy, 95% CI [2.45, 4.15]. Further, greater body dissatisfaction predicted higher levels of impaired mother-infant bonding over the first 6 months postpartum, 95% CI [.01, .48]. In turn, bonding difficulties predicted greater socioemotional dysfunction for infants at 1 year of age, 95% CI [.08, .79]. The overall indirect effect of body surveillance on infant socioemotional dysfunction through maternal body dissatisfaction and impaired mother-infant bonding was significant, 95% CI [.03, 1.00].

### Integrated Model With Depression And Body Dissatisfaction As Parallel Mediators

Finally, we tested an integrated model with general depression and body dissatisfaction as parallel mediators in a larger serial mediation model. We covaried the residuals of general depression and body dissatisfaction as they are both dimensions of maternal mental health. Full model results are reported in Table 2 and Fig. 2c. Greater body surveillance was associated with greater maternal depression, 95% CI [1.26, 4.11], and body dissatisfaction during pregnancy, 95% CI [2.44, 4.15]. Further, greater maternal depression associated with body surveillance predicted higher levels of impaired mother-infant bonding over the first 6 months postpartum, controlling for body dissatisfaction, 95% CI [.02, .30]. In turn, bonding difficulties predicted socioemotional dysfunction for infants at 1 year of age, 95% CI [.04, .76]. The overall indirect effect of body surveillance on infant socioemotional dysfunction at 1-year postpartum through maternal general depression and impaired mother-infant bonding was significant, 95% CI [.03, .55]. Notably, when controlling for depression, body dissatisfaction was no longer a significant mechanism through which body surveillance impacted mother-infant bonding and infant socioemotional dysfunction.

## Discussion

Living in a culture of persistent objectification, women may self-objectify and experience societal pressure to modify their bodies to achieve the thin ideal. During pregnancy, a period in which the body undergoes rapid changes to support fetal development, women who have internalized these messages and engage in more body surveillance may be at increased risk for negative mental health consequences, including body dissatisfaction and depression (Beech et al., 2020; Brock et al., 2021; Rubin & Steinberg, 2011). Maternal mental health, in turn, can undermine the mother-infant relationship and infant socioemotional functioning (McNamara et al., 2019; Slomian et al., 2019). By integrating research and theory in the areas of objectification and maternal-infant health, we found support for a novel conceptual framework in which self-objectification during pregnancy, as manifested by body surveillance, contributes to impaired mother-infant bonding and infant socioemotional functioning at 1-year postpartum through maternal mental health difficulties during pregnancy (i.e., body dissatisfaction and depression). Specifically, we found that mothers who endorsed higher levels of body surveillance also reported higher levels of depressive symptoms and body dissatisfaction during pregnancy. In turn, depressive symptoms and body dissatisfaction were associated with greater mother-infant bonding impairments during the 6 months following childbirth, which contributed to subsequent infant socioemotional dysfunction at 1-year postpartum (i.e., difficulties self-soothing, feeding, and sleeping).

When examining maternal depressive symptoms and body dissatisfaction during pregnancy as parallel mechanisms, results suggested that maternal depressive symptoms uniquely contribute to bonding impairments and subsequent maladjustment. Thus, maternal prenatal depression, which was moderately correlated with body dissatisfaction, might be a particularly salient pathway through which body surveillance undermines bonding and infant development. A potential explanation for this finding is that body dissatisfaction during pregnancy could be a prodromal symptom of an underlying mood disorder (Chan et al., 2020; Roomruangwong et al., 2017) or a risk factor for elevations in prenatal depression (Riquin et al., 2019). Indeed, a recent study found that risk of perinatal depression was four times higher in women dissatisfied with their body image (Riquin et al., 2019). Ultimately, results from the present study suggest that persistent depressed mood might be more detrimental to mother-infant bonding (e.g., by undermining maternal motivation and leading to disengagement) than unique aspects of body dissatisfaction. Nonetheless, given that it might contribute to risk for depression, body dissatisfaction remains an important target for investigations of prenatal mental health, particularly in perinatal research pursued within an objectification framework.

### Theoretical Implications

The present work makes several theoretical contributions to the literature on objectification. First, while pregnancy is a time when women may experience greater objectification and related consequences due to bodily changes, only a handful of studies (e.g., Rubin & Steinberg, 2011; Brock et al., 2021) have examined body surveillance, body dissatisfaction, and depression during this period. Thus, this study adds to limited research demonstrating that objectification theory, as originally posited by Fredrickson and Roberts (1997) and expanded over the years (Roberts et al., 2018) also applies to pregnant women. Second, to our knowledge, the present study is the first research to link body surveillance to the early mother-infant relationship and infant socioemotional functioning via prenatal depression and body dissatisfaction. While extant research has revealed an association between body surveillance and related mental health outcomes among mothers and children, no research to date has linked these variables during infancy. Further, results isolate a key developmental cascade in which maternal mental health during pregnancy, *prior to the birth of the child*, predicts early parenting behaviors and infant socioemotional functioning. Researchers increasingly recognize the pregnancy-postpartum transition as a critical window for intervention and assert that this “may be the most important way to ensure healthy child development” (Saxbe et al., 2018). Thus, prenatal maternal mental health represents a critical target for reducing risk for infant socioemotional difficulties, and results of the present study identify features of maternal mental health that have received limited attention in past research (i.e., body surveillance and body dissatisfaction) yet appear to have important implications for infant development.

### Limitations And Future Research Directions

It is important to acknowledge that the sample was comprised of women in committed relationships with men; participants also primarily identified as White and were from middle-class backgrounds, thereby limiting the generalizability of the results. There is a need for research examining objectification theory among more diverse populations (e.g., among sexual, gender, and racial minorities). For example, people of color, as well as sexual and gender minorities, experience unique forms of objectification, such as racialized sexual objectification and body policing (Flores et al., 2018). These additional forms of objectification may place pregnant people at even greater risk for self-objectification and related adverse mental health outcomes. There is also increasing recognition that researchers and clinicians alike must broaden their conceptualizations of pregnancy to include the experiences of not only cisgender women, but also transgender and nonbinary individuals (Moseson et al., 2020; Roosevelt et al., 2021).

There were also limitations to our measurement approach. First, while the present work examined body surveillance as a manifestation of self-objectification and downstream consequences identified by objectification theory (e.g., body dissatisfaction, depression, Roberts et al., 2018) as well as novel consequences (e.g., infant outcomes), some aspects of the objectification model remain untested in pregnant women. We did not measure specific types of objectification that pregnant women may experience, such as objectification directed at their size and shape (e.g., because their bodies no longer conform to feminine ideals of thinness) or involving denial of autonomy and subjectivity (e.g., because their bodies become public property). Relatedly, we only included one indicator of self-objectification. Thus, future research should examine how other indicators of self-objectification, such as internalized objectifying views (Noll & Fredrickson, 1998) and beliefs (Lindner et al., 2017), as well as non-bodily indicators of self-objectification (e.g., feeling invisible or lacking autonomy; Talmon & Ginzburg, 2016), might impact infant socioemotional functioning. To conduct this important work, measures that Specifically assess objectification and self-objectification in pregnant women will need to be developed and validated.

Second, our measure of body dissatisfaction was not Specifically designed for pregnancy and therefore may not capture specific appearance-related concerns associated with pregnancy (e.g., stretch marks, having a prototypical “baby bump”). Future research should consider newly developed measures, such as the Body Understanding Measure for Pregnancy Scale (BUMPs; Kirk & Preston, 2019) or the Body Experience during Pregnancy Scale (BEPS; Talmon & Ginzburg, 2018), that measure other facets of the body experience during pregnancy (e.g., body agency, estrangement, and visibility; satisfaction with appearing pregnant; weight gain concerns; physical burdens of pregnancy). Third, all data were collected using self-report questionnaires, raising the possibility of shared method bias. Although most objectification research has relied on self-report measures, there is increasing evidence that innovative approaches, such as eye tracking technology, can be utilized to assess the objectifying gaze, which may contribute to self-objectification (Gervais et al., 2013; Karsay et al., 2018). Fourth, measures of body surveillance, body dissatisfaction, and depression were gathered at the same time point. Although objectification theory posits that body surveillance contributes to subsequent body dissatisfaction and depression (Fredrickson & Roberts, 1997), it is also possible that maternal depression contributes to increased body surveillance. Thus, future studies examining these constructs at different time points across pregnancy are necessary to establish causality.

Finally, other factors of potential relevance to the study aims warrant attention in future research. For example, we did not examine the impact of objective measures of weight, such as pre-pregnancy and pregnancy body-mass index (BMI) and gestational weight gain, on body surveillance, general depression, and body dissatisfaction during pregnancy. Research on this topic is particularly important given that pregnant people with higher BMI are more likely to experience weight stigma (Mulherin et al., 2013; Parker & Pausé, 2018), which has the potential to exacerbate maternal prenatal mental health concerns and, in turn, infant outcomes. In addition, given that pregnancy and childbirth experiences may contribute to bonding impairments (e.g., Hanko et al., 2020; Sockol et al., 2014), research examining whether perinatal complications moderate the associations between prenatal body surveillance, body dissatisfaction, depression, and mother-infant bonding during the postpartum is warranted.

### Practice Implications

The present study sheds light on the importance of early interventions targeting not only maternal prenatal depression, but also body surveillance and dissatisfaction, to promote healthy infant development. Because body surveillance is a consequence of living in a culture that persistently objectifies women’s bodies, prevention efforts must begin at the societal level, long before people become pregnant. For instance, media campaigns can raise awareness of the insidious nature of valuing the appearance of girls and women over their other attributes and can help change perceptions of beauty by promoting body positivity and acceptance (e.g., #AerieReal and Dove’s Real Beauty campaign). Specific efforts to target objectification during pregnancy and the postpartum period are also warranted, such as campaigns promoting real images of mothers and their infants. For example, Mothercare’s #BodyProudMums is aimed at normalizing and celebrating the diversity and beauty of post-baby bodies. Broader dissemination of campaigns of this nature has the potential to promote maternal well-being. Finally, emerging evidence suggests that social media can be leveraged for the delivery of brief interventions to improve maternal body image and wellbeing (Wallis et al., 2021).

Unfortunately, societal change is slow, and objectification continues to manifest in ways that justify the patriarchy despite collective advances (Roberts et al., 2018). Therefore, beyond broad prevention efforts, there is also a need for targeted interventions informed by careful screening. Providers who interact regularly with pregnant women (e.g., obstetricians, nurses, midwives) could screen for elevations in body surveillance and associated body dissatisfaction and, when indicated, deliver brief interventions to disrupt self-objectification by promoting embodiment, which emphasizes positive self-talk, body functionality and agency, and experiencing the body from a subjective position rather than viewing themselves as sexual objects (Piran, 2017). It is critical that providers avoid protective paternalism and benevolent sexism discourses (e.g., restricting women’s behaviors during pregnancy to protect the fetus; Sutton et al., 2011). Instead, providers should counteract societal objectification and related self-objectification in ways that normalize the experience of body surveillance and body dissatisfaction during pregnancy and empower expectant mothers to prioritize their own mental health. Indeed, research suggests that pregnant women may be especially motivated to make behavioral changes that promote maternal and infant health (Ayyala et al., 2020); thus, pregnancy may be a promising developmental window for the delivery of interventions targeting self-objectification, body surveillance, and the cascade of negative mental health outcomes.

Additionally, despite the prevalence and underdiagnosis of perinatal depression (Cox et al., 2016; Underwood et al., 2016), there continues to be a critical need for universal screening and multidisciplinary approaches to maternal mental health care from a range of providers (e.g., obstetrics and gynecology, family medicine, and pediatric care providers; Muzik & Borovska, 2011) assessing multiple indicators of risk. For example, results highlight the utility of screening for body surveillance and dissatisfaction as an early manifestation of depressive symptoms, which can be done briefly and as part of routine prenatal care (Riquin et al., 2019; Stunkard et al., 1983). Further, perinatal depression screening can be effectively implemented by health and social service professionals with limited background in mental health (Segre et al., 2011). Professionals who come in regular contact with pregnant women (e.g., physicians, social workers, nurses) but do not have formal training in the assessment of depression could facilitate discussions of how women are relating to their bodies as they change throughout pregnancy and the postpartum. This approach has the potential to identify women who would benefit from intervention but might otherwise be overlooked by current screening practices.

More generally, our results suggest that doctors and clinicians might benefit from a broader conceptualization of maternal mental health during pregnancy including other dimensions of the perinatal experience, such as body shame and dissatisfaction. Women are routinely weighed throughout pregnancy for important medical reasons (e.g., to monitor fetal growth); however, routine weight assessments have the potential to increase body surveillance and adversely impact perinatal mental health. Thus, healthcare providers might consider approaching conversations about weight with sensitivity and with the goal of promoting a healthy pregnancy and baby. For instance, the National Institute of Child and Human Development’s *Pregnancy for Every Body* initiative aims to help people of all sizes achieve a healthy pregnancy (National Institute of Child and Human Development, 2019). In addition, providing psychoeducation on the natural bodily changes that occur across pregnancy may help mothers adjust to changing body ideals (Beech et al., 2020). By emphasizing body functionality, maternal healthcare providers may help women shift their focus away from their appearance-related concerns (Alleva & Tylka, 2021; Beech et al., 2020).

Finally, interventions for pregnant couples that seek to increase partner support may be particularly beneficial given evidence that partners may play a unique role in enhancing maternal body satisfaction during pregnancy (Watson et al., 2016). Indeed, given evidence that intimate partner humanization during pregnancy is associated with less body surveillance in mothers (Brock et al., 2021), it is important for interventions targeting self-objectification and its related consequences to include not only pregnant women, but also their partners.

## Conclusion

The present study demonstrated that body surveillance during pregnancy impacts infant socioemotional functioning at 1-year postpartum through increased prenatal depressive symptoms and body dissatisfaction and impaired mother-bonding during the 6 months following childbirth. Further, results suggested that maternal depressive symptoms may uniquely contribute to bonding impairments and subsequent child outcomes. This work expands on the limited body of research applying objectification theory to the experience of pregnancy and childbirth and supports a novel conceptual framework within which maternal self-objectification, manifested as body surveillance during pregnancy, impacts infant development as early as 1-year postpartum. Results highlight the potential utility of prenatal interventions guided by objectification theory to reduce the consequences of sexual objectification on mothers and their children.

## Figures and Tables

**Table 1:** Descriptive Statistics and Correlations

	1	2	3	4	5
1. Body Surveillance	1.00				
2. General Depression	**0.30** ***	1.00			
3. Body Dissatisfaction	**0.54** ***	**0.34** ***	1.00		
4. Impaired Bonding	**0.16** *	**0.26** ***	**0.23** **	1.00	
5. Infant Socioemotional Dysfunction	0.10	0.13	0.09	**0.21** **	1.00
Mean	3.67	37.90	9.34	8.75	29.41
SD	0.97	8.54	5.91	7.06	15.07
N	156	157	157	142	121

*Note.* Significant correlations are bolded.

* *p* < .05.

** *p* < .01.

*** *p* < .001.

**Table 2:** Path Analyses Examining the Impact of Body Surveillance, General Depression, and Body Dissatisfaction During Pregnancy on Infant Socioemotional Dysfunction via Impaired Bonding

	Unstandardized Estimate	95% CI^a^	Standardized Estimate
Model 1: General depression			
*Outcome*: Infant socioemotional dysfunction (ASQ), *R^2^* = 0.06			
**Impaired bonding (BOND)**	**0.40**	**[.04, .77]**	**0.19**
General depression (DEP)	0.11	[−.24, .43]	0.06
Body surveillance (OBJ)	0.87	[−2.34, 3.87]	0.06
*Outcome*: Impaired bonding (BOND), *R^2^* = 0.11			
**General depression (DEP)**	**0.18**	**[.05, .32]**	**0.22**
Body surveillance (OBJ)	0.80	[−.35, 2.00]	0.11
*Outcome*: General depression (DEP), *R^2^* = 0.11			
**Body surveillance (OBJ)**	**2.70**	**[1.29, 4.12]**	**0.31**
*Indirect effects*			
OBJ → DEP → ASQ	0.30	[−.64, 1.26]	
OBJ → BOND → ASQ	0.32	[−.07, 1.09]	
OBJ → DEP → BOND → ASQ	**0.19**	**[.04, .59]**	
Model 2: Body dissatisfaction			
*Outcome*: Infant socioemotional dysfunction (ASQ), *R^2^* = 0.05			
**Impaired bonding (BOND)**	**0.42**	**[.08, .79]**	**0.20**
Body dissatisfaction (BODY)	0.06	[−.51, .65]	0.03
Body surveillance (OBJ)	0.76	[−3.08, 4.16]	0.05
*Outcome*: Impaired bonding (BOND), *R^2^* = 0.10			
**Body dissatisfaction (BODY)**	**0.24**	**[.01, .48]**	**0.20**
Body surveillance (OBJ)	0.47	[−.81, 1.82]	0.06
*Outcome*: Body dissatisfaction (BODY), *R^2^* = 0.30			
**Body surveillance (OBJ)**	**3.30**	**[2.45, 4.15]**	**0.54**
*Indirect effects*			
OBJ → BODY → ASQ	0.21	[−1.69, 2.24]	
OBJ → BOND → ASQ	0.20	[−.29, 1.02]	
**OBJ → BODY → BOND → ASQ**	**0.34**	**[.03, 1.00]**	
Model 3: Integrated model			
*Outcome*: Infant socioemotional dysfunction (ASQ), *R^2^* = 0.05			
**Impaired bonding (BOND)**	**0.40**	**104, .76]**	**0.19**
General depression (DEP)	0.12	[−.25, .46]	0.07
Body dissatisfaction (BODY)	0.02	[−.62, .64]	0.01
Body surveillance (OBJ)	0.65	[−3.28, 3.97]	0.04
*Outcome*: Impaired bonding (BOND), *R^2^* = 0.13			
**General depression (DEP)**	**0.15**	**[.02, .30]**	**0.18**
Body dissatisfaction (BODY)	0.18	[−.07, .42]	0.15
Body surveillance (OBJ)	0.30	[−.97, 1.67]	0.04
*Outcome*: General depression (DEP), *R^2^* = 0.11			
**Body surveillance (OBJ)**	**2.68**	**[1.26, 4.11]**	**0.31**
*Outcome*: Body dissatisfaction (BODY), *R^2^* = 0.30			
**Body surveillance (OBJ)**	**3.30**	**[2.44, 4.15]**	**0.54**
*Indirect effects*			
OBJ → DEP → ASQ	0.31	[−.68, 1.33]	
OBJ → BODY → ASQ	0.05	[−2.01, 2.16]	
OBJ → BOND → ASQ	0.12	[−.37, .89]	
**OBJ → DEP → BOND → ASQ**	**0.16**	**[.03, .55]**	
OBJ → BODY → BOND → ASQ	0.24	[−.03, .83]	

*Note.* Significant parameters are bolded.

a 95% confidence intervals based on 10,000 bootstrapped samples.

## Data Availability

This study complied with Transparency and Openness Promotion (TOP) Guidelines. The study PI, Rebecca L. Brock (rebecca.brock@unl.edu), should be contacted to request access to research materials, analysis code, and data. Data management and analysis procedures for this project are registered at https://osf.io/hprk8, and we made no deviations from that plan.
